# Role of Ciminalum-4-thiazolidinone Hybrids in Molecular NF-κB Dependent Pathways

**DOI:** 10.3390/ijms25137329

**Published:** 2024-07-03

**Authors:** Dominika Szlachcikowska, Anna Tabęcka-Łonczyńska, Serhii Holota, Olexandra Roman, Yulia Shepeta, Roman Lesyk, Konrad A. Szychowski

**Affiliations:** 1 Department of Biotechnology and Cell Biology, Medical College, University of Information Technology and Management in Rzeszow, Sucharskiego 2, 35-225 Rzeszow, Poland; dszlachcikowska@wsiz.edu.pl (D.S.); rlesyk@wsiz.edu.pl (R.L.); kszychowski@wsiz.edu.pl (K.A.S.); 2 Department of Pharmaceutical, Organic and Bioorganic Chemistry, Danylo Halytsky Lviv National Medical University, Pekarska 69, 79010 Lviv, Ukraine; golota_serg@yahoo.com (S.H.); lesia_roman@ukr.net (O.R.); 3 Department of Organic Chemistry and Pharmacy, Lesya Ukrainka Volyn National University, Volya Avenue 13, 43025 Lutsk, Ukraine; 4 Department of Pharmaceutical Chemistry, National Pirogov Memorial Medical University, Pirogov 56, 21018 Vinnytsia, Ukraine; shepeta.yulia@gmail.com

**Keywords:** 4-thiazolidinones, A549, inflammation, fibroblasts, cytoskeleton

## Abstract

A range of hybrid molecules incorporating the ciminalum moiety in the thiazolidinone ring demonstrate significant anticancer and antimicrobial properties. Therefore, the aim of our study was to evaluate the properties and mechanism of action of two 4-thiazolidinone-based derivatives, i.e., 3-{5-[(Z,2Z)-2-chloro-3-(4-nitrophenyl)-2-propenylidene]-4-oxo-2-thioxothiazolidin-3-yl}propanoic acid (Les-45) and 5-[2-chloro-3-(4-nitrophenyl)-2-propenylidene]-2-(3-hydroxyphenylamino)thiazol-4(5H)-one (Les-247). In our study, we analyzed the impact of Les-45 and Les-247 on metabolic activity, caspase-3 activity, and the expression of genes and proteins related to inflammatory and antioxidant defenses and cytoskeleton rearrangement in healthy human fibroblasts (BJ) and a human lung carcinoma cell line (A549). The cells were exposed to increasing concentrations (1 nM to 100 μM) of the studied compounds for 24 h and 48 h. A decrease in the metabolic activity in the BJ and A549 cell lines was induced by both compounds at a concentration range from 10 to 100 µM. Both compounds decreased the mRNA expression of *NRF2* (nuclear factor erythroid 2-related factor 2) and *β-actin* in the BJ cells. Interestingly, a significant decrease in the level of *NF-κB* gene and protein expression was detected in the BJ cell line, suggesting a direct impact of the studied compounds on the inhibition of inflammation. However, more studies are needed due to the ability of Les-45 and Les-247 to interfere with the tubulin/actin cytoskeleton, i.e., a critical system existing in eukaryotic cells.

## 1. Introduction

Cancer is one of the most complex and multifaceted challenges to human health worldwide, necessitating continuous efforts to discover novel and effective therapeutic agents. Among the vast array of chemical compounds investigated for their potential anticancer properties, 4-thiazolidinones, a class of heterocyclic compounds, have emerged as promising candidates [1,2]. Heterocycles, particularly 4-thiazolidinone derivatives, have garnered significant attention in medicinal chemistry due to their diverse biological activities [3]. These activities include antimicrobial [4,5], antiviral [6], antiproliferative [7], anti-inflammatory [8], anti-tuberculosis [9], antidiabetic [10], antihypertensive [11], neuroprotective [12], and anticancer properties [13,14]. Numerous reports not only describe their biological activities but also provide comprehensive insights into their mechanisms of action and specific biological targets associated with crucial biochemical processes, such as tumor cell growth, mitosis, the life cycle of microorganisms, the progression of inflammatory conditions, and the early onset of type II diabetes mellitus (T2D) [15]. To date, it has been described that 4-thiazolidinones exert their mechanisms of action primarily through the activation of peroxisome proliferator-activated receptors (PPAR), especially PPARγ, which is a nuclear receptor involved in the regulation of various metabolic processes [16]. PPARγ activation regulates the expression of certain genes and proteins related to lipid metabolism, glucose homeostasis, and inflammation [17]. Moreover, 4-thiazolidinones enhance insulin sensitivity through PPARγ by promoting adipocyte differentiation and reducing adipose tissue inflammation [18]. This specific mechanism of action highlights the role of 4-thiazolidinones as PPARγ agonists, offering a targeted approach for managing metabolic disorders, e.g., T2D. Thus, the PPARγ agonists are currently gaining significant attention due to their beneficial therapeutic effects and the absence of typical adverse reactions [19].

**Figure 1:**
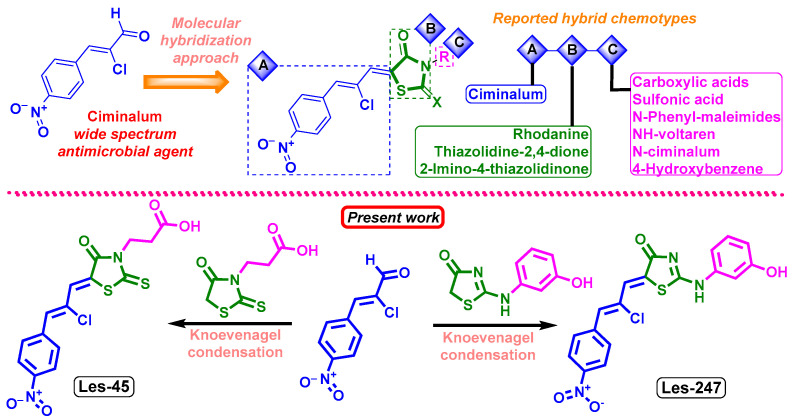
General scheme of the early reported diversity-oriented libraries of ciminalum-4-thiazolidinone hybrids, synthesis, and structures of hybrids Les-45 and Les-247.

Currently, there is a successful trend in the design of molecular structures with drug-like properties involving the “hybrid-pharmacophore” strategy, also known as molecular hybridization [1]. This approach combines various fragments from biologically active molecules or drugs into a single molecule, resulting in the formation of new chemical compounds with unique biological properties [20]. As reported, the 4-thiazolidinone core exhibits significant potential for chemical modifications, providing extensive opportunities for the development of novel derivatives with enhanced properties and diverse functionalities [21,22]. A promising trend in the development of potential antitumor agents containing the 4-thiazolidinone structure involves hybridization with a ciminalum (2-chloro-3-(4-nitrophenyl)-2-propenylidene) fragment (Figure 1). A range of hybrid molecules incorporating the ciminalum moiety in position 5 of the 4-thiazolidinone ring has been designed following the hybrid-pharmacophore approach and diversity-oriented synthesis.

It was reported that the ciminalum–thiazolidinone hybrid molecules 3-{5-[(Z,2Z)-2-chloro-3-(4-nitrophenyl)-2-propenylidene]-4-oxo-2-thioxothiazolidin-3-yl}propanoic acid (Les-45) and 5-[2-chloro-3-(4-nitrophenyl)-2-propenylidene]-2-(3-hydroxyphenylamino)thiazol-4(5H)-one (Les-247) showed a broad spectrum of growth inhibition activity against human tumor cells and some distinctive patterns of selectivity [23,24] (Figure 1). The cytotoxic effect of Les-45 was found in leukemia (MOLT-4, SR), colon cancer (SW-620), brain cancer (SF539), melanoma (SK-MEL-5), gastric cancer (AGS), human colon cancer (DLD-1), and breast cancer (MCF-7, MDA-MB-231) cell lines [23]. Hybrid Les-247 was reported to inhibit selectively the growth of leukemia (K562) and colon cancer (SW-620) cell lines, which is probably associated with immunosuppressive activity [24]. 

Therefore, to extend the knowledge of the potential mechanisms of action of ciminalum–thiazolidinone hybrid molecules Les-45 and Les-247, we evaluated their cytotoxicity, effect on metabolic activity, caspase-3 activity, impact on the expression of genes and proteins involved in the inflammatory response, antioxidant defenses, and cytoskeleton rearrangement processes in healthy human fibroblasts (BJ) and the human lung carcinoma cell line (A549).

## 2. Results

### 2.1. Synthesis of Ciminalum-4-thiazolidinone Hybrids Les-45 and Les-247

The synthesis of the studied hybrids Les-45 and Les-247 was easily performed in the conditions of Knoevenagel condensation of ciminalum with 3-(4-oxo-2-thioxothiazolidin-3-yl)propanoic acid and 2-((3-hydroxyphenyl)amino)thiazol-4(5H)-one, respectively, as presented in Figure 1. The compounds were obtained with sufficient yields and purity. Detailed synthetic procedures and characterization of the properties of the compounds were reported in [23] for compound Les-45 and in [24] for derivative Les-247. 

### 2.2. Resazurin Reduction Assay

**Figure 2:**
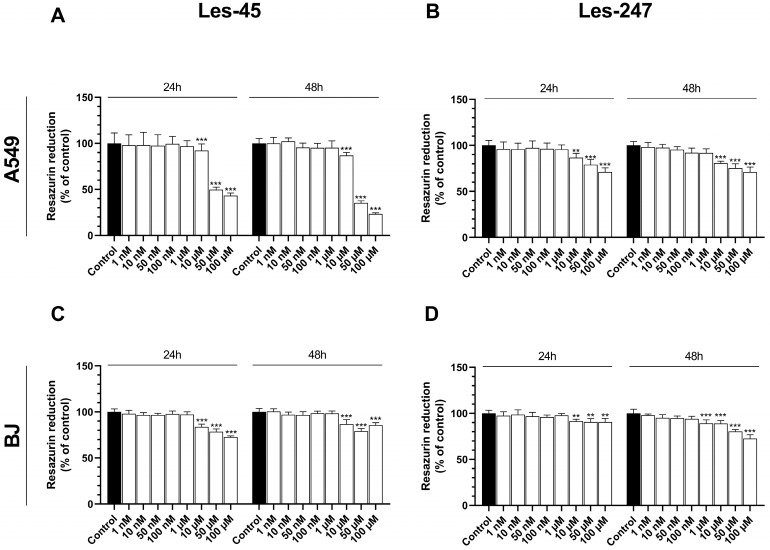
Resazurin cell viability assay. Effect of increasing concentrations of Les-45 and Les-247 (1 nM–100 μM) on the metabolic activity in the A549 (**A**,**B**) and BJ (**C**,**D**) cell lines after the 24- and 48-h exposures. Data are expressed as a mean (*n* = 6), with standard deviation. Statistically significant values determined by Tukey’s test for each study group. ** *p* < 0.01, *** *p* < 0.001, compared with control cells.

Les-45 significantly decreased the metabolic activity of A549 at the concentrations of 10, 50, and 100 µM after both the 24-h and 48-h treatments (by 7.76, 50.17, and 56.84%, respectively, at the 24-h exposure and 13.25, 64.67, and 76.60%, respectively, at the 48-h exposure, compared to the control) (Figure 2A). After the 24-h treatment of the BJ cell line, Les-45 decreased the resazurin reduction at the concentrations of 10, 50, and 100 µM (by 16.33, 21.59, and 27.33%, respectively), compared to the control (Figure 2C). Similarly, after the 48-h exposure of the BJ cells to 10 µM, 50 µM, and 100 µM of Les-45, the cell metabolism decreased by 13.23, 21.00, and 14.29%, respectively, compared to the control (Figure 2C). Similarly, the IC_50_ values increased during the Les-45 exposure, reaching 50.05 µM and 33.28 µM after 24 h and 48 h, respectively (Table 1). 

Compound Les-247 decreased A549 cell metabolism after the 24-h treatment with 10, 50, and 100 µM by 13.38, 20.94, and 28.89%, respectively, compared to the control (Figure 2B). In turn, after the 48-h treatment of the A549 cells with 10, 50, and 100 µM of Les-247, the metabolic activity decreased by 1.19, 24.73, and 29.04%, respectively, compared to the control (Figure 2B). In the BJ cell line exposed to the action of Les-247, the metabolic activity was decreased only in the concentration range of 10–100 µM (from 8.55 to 9.49%) in comparison to the control (Figure 2D). However, the 48-h treatment with Les-247 in the concentration range between 1 and 100 µM caused a decrease in BJ metabolic activity (from 10.84 to 27.34%, compared to the control) (Figure 2D).

### 2.3. Effect of the Tested Compounds on the LDH Release Level

**Figure 3:**
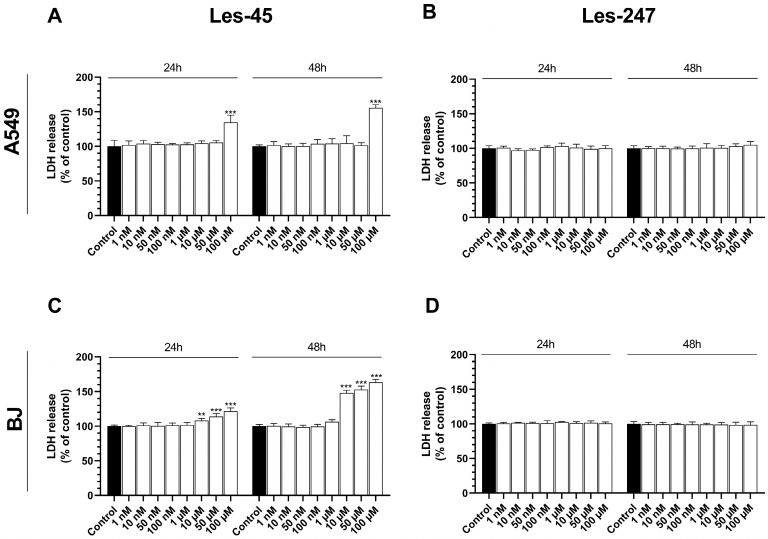
Activity of lactate dehydrogenase. Effect of increasing concentrations of Les-45 and Les-247 (1 nM–100 μM) on the level of LDH release in the A549 (**A**,**B**) and BJ (**C**,**D**) cell lines after the 24- and 48-h exposures. Data are expressed as a mean (*n* = 6), with standard deviation. Statistically significant values determined by Tukey’s test for each study group. ** *p* < 0.01, *** *p* < 0.001, compared with control cells.

After the 24-h exposure of the A549 cells to the increasing concentrations of Les-45, only the 100 µM concentration increased the LDH release by 34.79%, compared to the control (Figure 3A). Similarly, after the 48-h exposure to Les-45, only the 100 µM dose of the compound increased the LDH release level by 55.72%, compared to the control (Figure 3A). The 24-h exposure of the BJ cell line to Les-45 caused an increase in the LDH release at the 10, 50, and 100 µM concentrations (by 8.14, 13.85, and 21.57%, respectively) relative to the control (Figure 3C). Similarly, after the 48-h exposure, an increase in the LDH release was observed at the 10, 50, and 100 µM concentrations (by 48.09, 52.84, and 63.44%, respectively) in comparison to the control (Figure 3C). After the 24-h and 48-h exposures to Les-247, no LDH release was observed in both studied cell lines at any of the concentrations applied (Figure 3B,D).

### 2.4. Caspase-3 Activity

**Figure 4:**
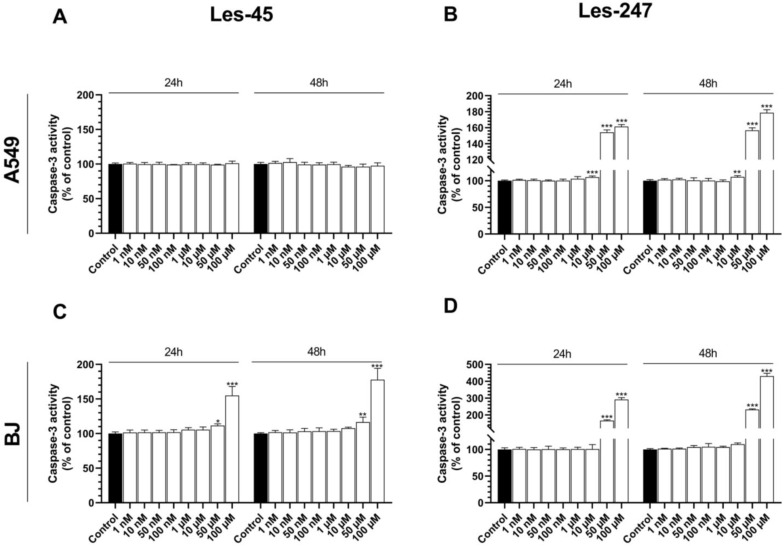
Caspase-3 activation. Effect of increasing concentrations of Les-45 and Les-247 (1 nM–100 μM) on caspase-3 activity in the A549 (**A**,**B**) and BJ (**C**,**D**) cell lines after the 24- and 48-h exposures. Data are expressed as a mean (*n* = 6) with standard deviation. Statistically significant values determined by Tukey’s test for each study group. * *p* < 0.05, ** *p* < 0.01, *** *p* < 0.001, compared with control cells.

Les-45 did not influence caspase-3 activity in the A549 cell line at any concentration of the compound after the 24-h and 48-h treatments in comparison to the control (Figure 4A). After the 24-h exposure of the BJ cells to Les-45, an increase in caspase-3 activity at the concentrations of 50 and 100 µM was observed (by 11.55 and 55.08%, respectively), compared to the control (Figure 4C). After the 48-h treatment of the BJ cells with 50 µM and 100 µM of Les-45, the activity of caspase-3 increased by 16.59 and 77.87%, respectively, compared to the control (Figure 4C). 

After the 24-h exposure to 10, 50, and 100 µM of Les-247, caspase-3 activity increased substantially in the A549 cells (by 6.69, 54.21, and 61.43% respectively), compared to the control (Figure 4B). A similarly high increase was noted after 48 h (by 7.20, 56.71, and 78.80%, respectively), compared to the control at the 10, 50, and 100 μM concentrations (Figure 4B). The most intensive increase in caspase-3 activity induced by Les-247 was observed in the BJ cell line at the two highest micromolar concentrations of 50 and 100 μM. After the 24-h exposure of the BJ cell line to Les-247, the activity of caspase-3 increased by 66.23 and 191.75%, respectively, compared to the control (Figure 4D). Similarly, the 48-h treatment with 50 and 100 µM increased caspase-3 activity by 131.87 and 330.28%, respectively, compared to the control (Figure 4D).

### 2.5. Fluorescence Microscope Analysis

**Figure 5:**
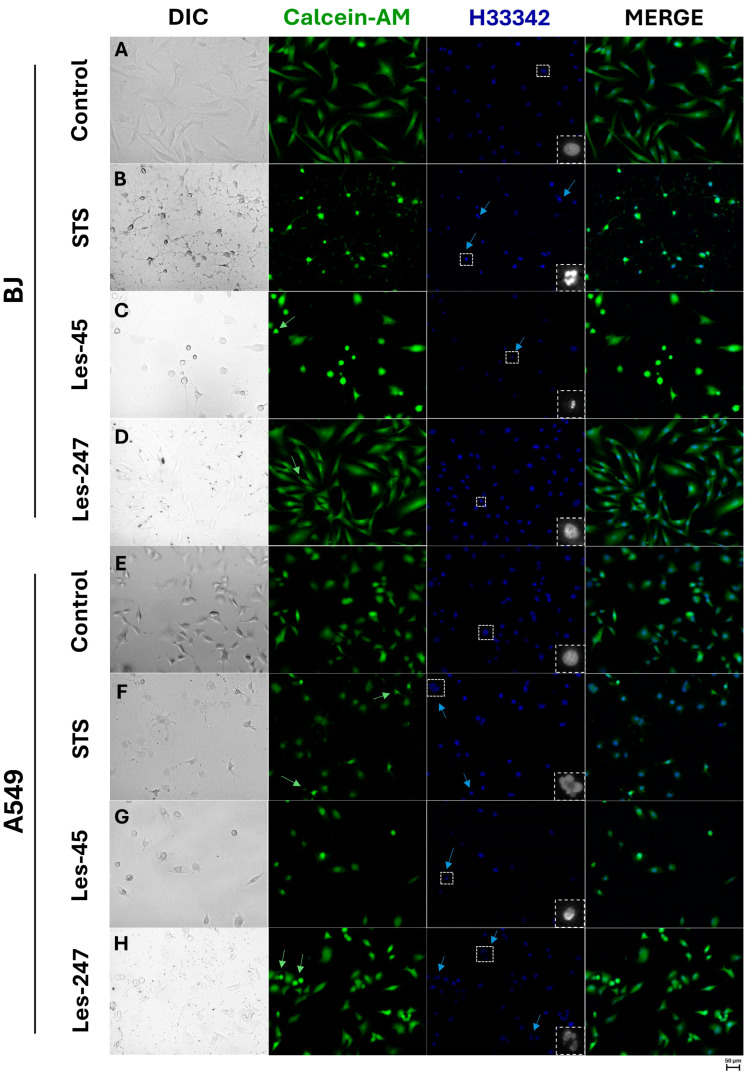
Confocal imaging with Hoechst 33342 (H33342) and Calcein–AM staining of the BJ and A549 line cells after the exposure to 10 μM of Les-45 (**C**,**G**), 10 μM of Les-247 (**D**,**H**), 1 μM of staurosporine (STS) (**B**,**F**) and without the compounds (**A**,**E**) after 24 h treatment. The arrows mark apoptotic vesicles or apoptotic nuclei. The 100× magnification was used. Dashed squares indicate the regions used for the zoom.

Apoptotic cells are characterized by nuclear condensation and DNA fragmentation, detectable through staining with Hoechst 33342. Hoechst 33342 selectively binds to fragmented DNA in apoptotic bodies, resulting in blue fluorescence emission. In turn, living cells exhibit esterase activity, which can be visualized as green fluorescence through Calcein–AM staining. In the BJ cells, we observed the changes in cell morphology as well as the formation of apoptotic vesicles after the cell treatment with Les-45. Moreover, Les-45 caused a decrease in the number of cells in both A549 and BJ cell lines. In addition, the fluorescence microscope analysis revealed the formation of apoptotic bodies, membrane blebbing, and chromatin condensation in the A549 cells exposed to 10 μM of Les-247 (Figure 5). 

### 2.6. Real-Time PCR Analysis of mRNA Specific for Genes Encoding PPARγ, NF-κB, NRF2 and ACTB

**Figure 6:**
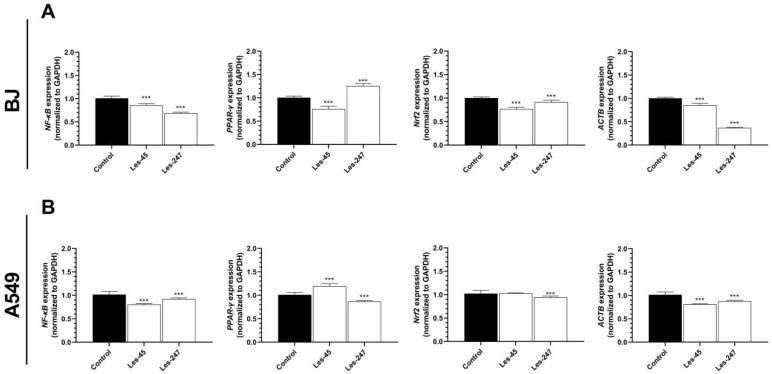
Gene expression analysis. Effect of 10 μM Les-45 and Les-247 on NF-κB, PPARγ, NRF2, and ACTB mRNA expression in the BJ (**A**) and A549 (**B**) cell lines after the 24-h exposure to the tested compounds. Data are expressed as a mean (*n* = 12) with standard deviation. Statistically significant values determined by Tukey’s test for each study group. *** *p* < 0.001, compared to the control cells.

The BJ cells treated with Les-45 for 24 h were characterized by a 15.12, 24.28, 23.29, and 14.70% decrease in the *NF-κB, PPARγ, NRF2*, and *ACTB* mRNA expression, respectively, compared to the control (Figure 6A). Similarly, Les-247 reduced the *NF-κB, NRF2*, and *ACTB* mRNA expressions by 32.05, 8.81, and 63.27%, respectively, compared to the control (Figure 6A). However, 10 µM of Les-247 increased the *PPARγ* mRNA expression by 25.03%, compared to the control (Figure 6A). 

The results of these experiments showed that the *NF-κB* and *ACTB* mRNA expression in the A549 cell line was decreased by 21.07% and 20.15% after the 24-h exposure to 10 µM of Les-45, respectively, compared to the control (Figure 6B). In turn, Les-45 increased the *PPARγ* mRNA expression by 18.22%, compared to the control cells and did not affect the *NRF2* mRNA expression (Figure 6B). In the A549 cells, Les-247 reduced the *NF-κB, PPARγ, NRF2*, and *ACTB* mRNA expression by 9.60, 14.00, 7.77, and 13.67%, respectively, compared to the control (Figure 6B). 

### 2.7. NF-κB, IκB-α, pIκB-α, STAT3, β-Tubulin, and β-Actin Proteins Expression

**Figure 7:**
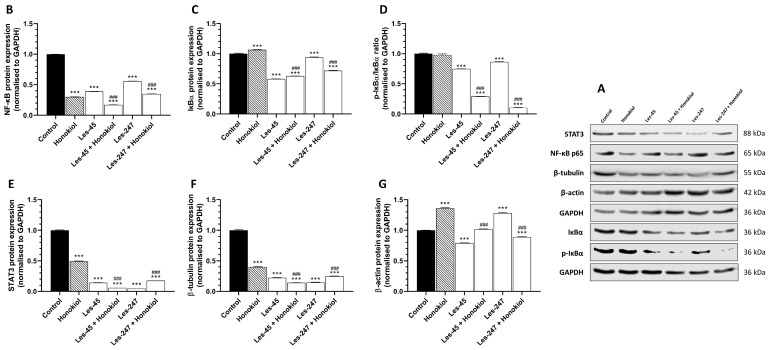
Western blot analysis of inflammation-related and cytoskeletal proteins. Proteins bands, as obtained by western blotting (**A**). Representative Western blots of NF-κB (**B**), STAT3 (**C**), IκB-α (**D**), pIκB-α (**E**), β-tubulin (**F**), and β-actin (**G**) levels in BJ cells treated with honokiol (15 µM), Les-45 (10 µM), cells co-treated with Les-45 (10 µM) and honokiol (15 µM), Les-247 (10 µM), and cells co-treated with Les-247 (10 μM) and honokiol (15 μM). Protein bands were quantified by densitometry. The results are shown as the percentage of protein relative to the control. Each column represents the mean ± SEM of three independent experiments. The blots were stripped and reprobed with anti-GAPDH antibody to control the amounts of protein loaded onto the gel. The statistical significance of each data point was analyzed by Turkey’s test, using one-way ANOVA for each group. *** *p* < 0.001, compared to the control group. ^###^ *p* < 0.001, compared to the honokiol-treated group.

After the 24-h exposure of the BJ cells to Les-45, we observed a downregulated level of NF-κB, IκB-α, pIκB-α, STAT3, β-tubulin, and β-actin expression by 60.98, 41.77, 25.13, 85.78, 77.81, and 21.12%, respectively, compared to the control (Figure 7B–G). A similar effect on NF-κB, IκB-α, pIκB-α, STAT3, and β-tubulin was observed in the Les-247 treated cells (44.00, 6.19, 13.75, 95.35, and 85.50% decrease, respectively) and those cotreated with Les-45 and honokiol (82.76, 37,00, 70.96, 94.16, and 85.79% decrease, respectively), compared to the control cells (Figure 7B–F). Les-45 in the co-treatment with honokiol did not affect the β-actin protein level in the BJ cell line (Figure 7G). An increase in the IκB-α and β-actin protein expression was found in the honokiol-treated BJ cells compared to the control cells (6.5 and 35.87% increase, respectively) (Figure 7D,G). An increased level of β-actin protein expression (by 28.06%) was also exhibited by the Les-247 treated cells, compared to the control (Figure 7G). In contrast, a decrease in the NF-κB, STAT3, and β-tubulin protein expression was demonstrated in the BJ cells after the exposure to honokiol (by 69.91, 50.51, and 60.28%, respectively), compared to the control cells (Figure 7B,C,F). Honokiol alone did not affect the pIκB-α protein level in the BJ cell line (Figure 7E).

**Figure 8:**
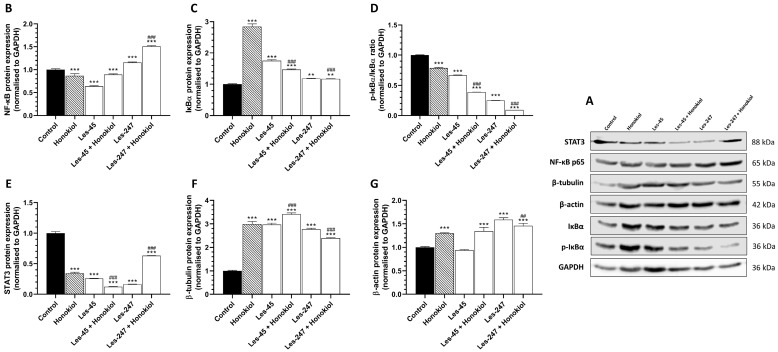
Western blot analysis of inflammation-related and cytoskeletal proteins. Proteins bands, as obtained by western blotting (**A**). Representative Western blots of NF-κB (**B**), STAT3 (**C**), IκB-α (**D**), pIκB-α (**E**), β-tubulin (**F**), and β-actin (**G**) levels in A549 cells treated with honokiol (15 µM), Les-45 (10 µM), cells co-treated with Les-45 (10 µM), honokiol (15 µM), and Les-247 (10 µM), and cells co-treated with Les-247 (10 μM) and honokiol (15 μM). Protein bands were quantified by densitometry. The results are shown as the percentage of protein relative to the control. Each column represents the mean ± SEM of three independent experiments. The blots were stripped and reprobed with anti-GAPDH antibody to control the amounts of protein loaded onto the gel. The statistical significance of each data point was analyzed by Turkey’s test, using one-way ANOVA for each group. ** *p* < 0.01, *** *p* < 0.001, compared to the control group. ^##^ *p* < 0.01, ^###^ *p* < 0.001, compared to the honokiol-treated group.

The Western blot analyses showed that the 24-h exposure to 10 μM Les-45, 10 μM Les-247, and 15 μM honokiol, as well as the Les-45 co-treatment with honokiol and the Les-247 co-treatment with honokiol, decreased the level of STAT3 (by 74.27, 83.58, 65.52, 87.52, and 36.96%, respectively) and pIκB-α (by 33.21, 75.00, 21.33, 61,27, and 90.81%, respectively) in the A549 cells, compared with the control cells (Figure 8C,E). The NF-κB protein expression decreased in the A549 cells treated with Les-45 (by 36.27%) and in the co-treatment with Les-45 and honokiol (by 10.59%), compared to the control cells (Figure 8B). In contrast, an increase in the NF-κB protein expression was observed in the Les-247 treatment (by 15.49%) and in the co-treatment with Les-247 and honokiol (by 50.44%) (Figure 8B). As expected, as an NF-κB inhibitor, honokiol downregulated the level of NF-κB in the human lung carcinoma cells by 13.19%, compared to the control (Figure 8B). These changes were associated with changes in IκB-α. An increase in the IκB-α protein expression was found in the A549 cells treated with Les-45 (by 75.16%), Les-247 (by 18.41%), Les-45 with honokiol (by 46.83%), Les-247 with honokiol (by 17.43%), and honokiol alone (by 183.93%), compared to the control cells (Figure 8D). A similar effect was observed for β-tubulin. An upregulated level of β-tubulin protein expression was observed after the exposure of the cells to Les-45 (by 196.82%), Les-247 (177.02%), Les-45 with honokiol (by 241.17%), Les-247 with honokiol (by 139.06%), and honokiol alone (by 198.51%), compared to the control cells (Figure 8F). The level of β-actin in A549 cells treated with Les-247, Les-247 with honokiol, Les-45 with honokiol, and honokiol alone was increased by 58.98, 45.91, 34.58, and 29.87%, respectively, compared to the control cells (Figure 8G). Les-45 did not influence the β-actin protein level in the A549 cells (Figure 8G).

## 3. Discussion

**Figure 9:**
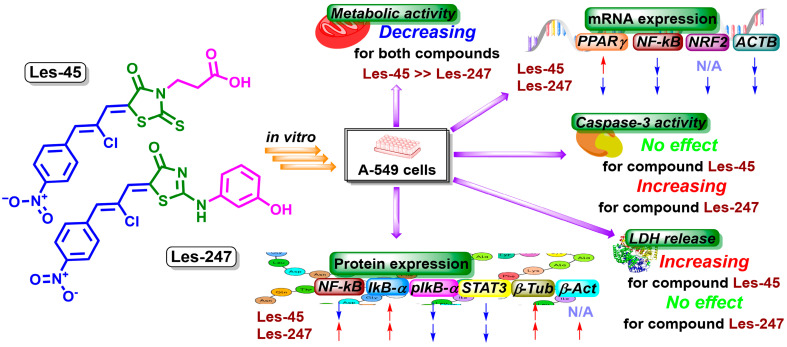
Schematic presentation of the cytotoxic effects and potential mechanisms of action of derivatives Les-45 and Les-247. N/A—no effect. Red arrows pointing upwards represent an increase in mRNA/protein expression. Blue arrows pointing down represents a decrease in mRNA/protein expression.

Investigations of biologically active heterocycles with a 4-thiazolidinone scaffold hold promise for advancing the development of novel anticancer compounds [20,25]. Here, we evaluated the effect of 4-thiazolidinone hybrids Les-45 and Les-247 on the viability and metabolic activity of human skin fibroblast (BJ) and human epithelial lung carcinoma (A549) cell lines (Figure 9). As shown by the resazurin reduction assay, both compounds decreased the metabolism of the BJ and A549 cells at the three highest µM concentrations after 24 and 48 h; therefore, the use of these compounds as a potent anticancer drug is limited. The resazurin reduction assay revealed that Les-45 decreased the metabolic activity/proliferation of the A549 cells more intensively than Les-247 (Figure 9). Nevertheless, Les-247 was found to significantly decrease cell metabolism in the BJ cell line already at the concentration of 1 μM after the 48-h exposure. It was previously reported that the half-maximal inhibitory concentration value (IC_50_) for the cytostatics cisplatin and doxorubicin against A549 lung cancer cells was determined to be 6.14 µM and 71 µM, respectively [26,27]. In this study, Les-45 inhibited the viability of the A549 cell line with the IC_50_ value ranging from 50.05 µM to 33.29 µM in the 24- and 48-h treatments. Additionally, the IC_50_ value for curcumin, a plant-derived polyphenolic compound known for its anticancer properties, against A549 lung cancer cells has been reported to be around 20 µM [28]. Previously, Buzun et al. [23] found that the studied ciminalum–thiazolidinone derivative Les-45 displayed antimitotic activity with mean GI_50_ values of 1.57 μM and a certain sensitivity range toward SF539, SK-MEL-5, MOLT-4, SW-620, AGS, DLD-1, MCF-7, MDA-MB-231, and U251 cancer cell lines [23]. Finiuk et al. [29] reported that ciminalum–4-thiazolidinone hybrids with phenyl-pyrrolidine-2,5-dione moieties demonstrated significant cytotoxic effects at the micromolar level against various cancer cell lines, including leukemia, colon cancer, central nervous system tumors, and ovarian cancer [29]. Additionally, previous research showed that Les-247 structural analogs containing a 2-chloro-3-(4-nitrophenyl)propenylidene substituent in combination with a 4-hydroxyphenylamino moiety exhibited one of the most potent activities with specific selectivity against certain cancer cell lines. It has also been reported that Les-247 selectively inhibits the growth of K562 and SW-620 cell lines, which is probably associated with immunosuppressive activity [24].

Therefore, to extend the knowledge of the potential mechanisms of action of the studied thiazolidinone derivatives, we evaluated their effect on caspase-3 activity, genes, and proteins whose expression is related to inflammation, antioxidant defenses, and cytoskeleton rearrangement. The anticancer properties of 4-TZDs (4-thiazolidinones) were observed to rely on the activation of caspase cascades [30,31]. One of the primary markers of apoptosis is the activation of caspase-3, which results in the degradation of chromosomal DNA into nucleosome-sized fragments and the degradation of the nuclear and cytoskeleton proteins, finally leading to membrane blebbing [32]. Our study showed that, after the 24-h exposure to Les-45, caspase-3 activity increased in the BJ cell line at the two highest micromolar concentrations. In turn, the treatment did not influence caspase-3 activity in the A549 cell line after the 24- and 48-h exposures (Figure 9). Cancer cells are often found to overexpress proteins that play important roles in resisting the activation of the apoptotic cascade [33]. Therefore, a better understanding of the molecular mechanisms underlying tumor resistance to apoptotic cell death is crucial for the development of molecular targeted therapies [34]. Additionally, our results of confocal microscopy analysis showed the ability of Les-247 to induce apoptotic-like changes in the nucleus in both tested cell lines. In our experiments, the increase in caspase-3 activity was observed in the Les-247 treatment at the 10–100 μM concentration range in the A549 cell line (Figure 9). However, the observation that Les-247 caused much stronger caspase-3 activation in the BJ cells is disturbing. A similar trend was reported for another 4-TZD derivative (Les-236) by Szychowski et al. [35]. Our results are consistent with the literature data. Previously, it was described that 4-TZDs may induce active apoptosis in both normal and cancer cell lines at a wide range of concentrations [35,36,37,38]. The discrepancies in the LDH release, resazurin reduction test, and caspase-3 assay result from the different mechanisms of action of Les-45 and Les-247. Specifically, the increase in the LDH release accompanied by a decrease in resazurin reduction suggests the toxicity of the Les-45 compound and the absence of apoptosis. In contrast, the decrease in resazurin reduction with an increase in caspase-3 activity indicates apoptotic cell death caused by Les-247. This relationship cannot be detected by the standard National Cancer Institute (NCI) protocol [23,24].

It has been reported that 4-TZD derivatives are potent and selective activators of PPARγ [39]. PPARγ maintains redox homeostasis through the activation and suppression of various signaling pathways [40]. Moreover, it plays a role in such processes as glucose and lipid metabolism, cell cycle regulation, apoptosis, and inflammation [41]. Our results showed that 10 μM of Les-45 decreased the level of expression of *NF-κB* and *ACTB* mRNA and simultaneously increased the level of expression of *PPARγ* mRNA in the A549 cells (Figure 9). To date, it has been reported that the activation of PPARγ leads to inhibition of the development of melanoma, colon, lung, and breast cancer cells in vitro [41,42,43]. On the other hand, in samples from human lung tumors, decreased expression of PPARγ was correlated with poor prognosis [44]. In this study, Les-247 significantly decreased the mRNA levels of all the tested genes in A549, including *PPARγ* (Figure 9). However, caution must be taken while interpreting data on these compounds because they are likely to engage multiple pathways distinct from PPARγ [45].

4-TZDs have been found to exhibit anti-inflammatory activity exerted inter alia through the *NF-κB* inhibition [46]. NF-κB is a family of structurally related transcription factors that play a major role in inflammation and immune responses [47]. Furthermore, NF-kB plays a pivotal role in tumor progression by inhibiting apoptosis and promoting cell proliferation [48]. Constitutive or abnormal activation of NF-κB is frequently observed in various solid tumors, such as lung cancer [49], ovarian cancer [50], prostate cancer [51], gastric carcinoma [52], colorectal cancer [53], and breast cancer [54]. In our experiments, both tested heterocycles decreased the *NF-κB* mRNA expression levels in the BJ and A549 cells. Moreover, it has been demonstrated that TZDs (pioglitazone, rosiglitazone), which are insulin-sensitizing medications used in T2D, decreased *NF-κB* mRNA in mouse lung tissue [55], mouse primary cholangiocytes [56], and human vascular endothelial cells (HUVEC) [57]. As reported by Lu et al. [46], 4-thiazolidinone-1,3,5-triazine hybrids induced potent in vitro inhibition of NF-κB protein activation in a murine macrophage cell line (RAW264.7) and, consequently, exerted a neuroprotective effect in mice through attenuation of inflammation, oxidative stress, and apoptosis [46]. Opposite results were obtained by Bar et al. [58], who revealed that 4-TZD treatment resulted in increased *NF-κB* mRNA expression [58].

The present results corroborate the crosstalk between PPARγ and NF-κB [59]. Reciprocal regulation between these genes has been shown. The anti-inflammatory properties of PPARs include the potential to interfere with transcriptional pathways involved in inflammatory responses, e.g., modulation of NF-κB signaling or promotion of the inactivation of NF-κB. Possible mechanisms of inactivation include direct binding and, consequently, inactivation of p65 NF-κB [60,61]. In our study, the attenuated mRNA and protein expression of *NF-κB* correlated with the increased *PPARγ* mRNA level in the A549 cell line after the treatment with Les-45 (Figure 9). A similar effect was observed in the Les-247 variant in the BJ cell line. The treatment of the A549 cells with these compounds inhibited the phosphorylation and degradation of IκBα, leading to a reduction in NF-κB DNA binding activity. This suggests that their anti-inflammatory effects may be due, at least in part, to the inhibition of NF-κB. The observed simultaneous ability of Les-247 to reduce the NF-kB level in the BJ cells and increase its level in the A549 cell line appears to be of interest since this is an upstream event. This effect was strengthened by the combined treatment with honokiol, i.e., an anti-inflammatory and antioxidant agent used in this study as a tool compound for evaluation of the NF-κB function in response to Les-45 and Les-247 due to its effective blocking of NF-κB activation [62]. In fact, the NF-kB reduction can decrease the levels of pro-inflammatory mediators, such as cytokines, chemokines, and various enzymes (such as matrix metalloproteinases or nitric oxide synthase), interfering with downstream signaling components crucial for inflammatory response and degenerative processes. However, we suppose that further investigations in this field need to be performed.

On the other hand, accumulating evidence supports the concept that NRF2 plays a key role in anti-inflammatory processes. The *NRF2* gene encodes a protein, which is a transcription factor that affects the expression of genes responsible for oxidative stress and inflammation prevention in mammalian cells. To date, studies have indicated that NRF2 exerts a negative regulatory influence on the NF-κB signaling pathway through multiple mechanisms [63]. For instance, as reported by Cho et al. [64], reduced expression of *NRF2* correlated with attenuated *PPARγ* levels in mouse lung cells [64]. However, we observed this trend in our study only for Les-45 in BJ and for Les-247 in the A549 cell line. On the other hand, the NRF2 and STAT3 signaling pathways can interact with each other, which undoubtedly increases the complexity of their signal transduction and the diversity of drug treatment targets [65]. Since crosstalk between STAT3 and NRF2 signaling may occur in various tumors, the specific mechanisms and functions must be determined to better guide clinical medication and the development of new drugs [66]. Unfortunately, the *NRF2* mRNA expression under treatment with 4-TZD derivatives has been poorly elucidated.

One of the possible mechanisms of anticancer activity exhibited by TZDs involves the inhibition of particular enzymes [67]. Among these microtubules are cytoskeletal elements known as drivers of directed cell migration and organelle trafficking, affecting cell shape, cell motility, and cell division [68]. Therefore, inhibition of tubulin polymerization leads to mitotic arrest and prevents cells from entering the critical S and M phases with damaged DNA [69]. Modern data are the reasons for the evaluation of different novel types of hybrids 4-thiazolidine-bearing molecules for the design of a potential tubulin inhibitor [13]. In order to investigate whether the decrease in β-actin observed within 24 h in the BJ and A549 cells treated with the investigated compounds was related to any changes in the cytoskeleton, the key cytoskeletal proteins were analyzed using Western blot analysis. Both compounds were able to interfere with the tubulin/actin cytoskeleton, a critical system existing in eukaryotic cells. The results presented herein show that β-actin was downregulated in the A549 cells at the transcriptional but not translational level after the treatment with Les-45 and Les-247. A massive decrease in the expression of certain genes is often correlated with an increase in the expression of the corresponding protein, which, in turn, acts as a negative regulator of the expression of this gene [70]. Indeed, the measured β-actin protein expression showed an increase in this parameter after the treatment with the analyzed compounds in the A549 cells. In turn, aberrant expression of isotypes of β-tubulins in cancer tissues has been reported to regulate metastasis, cancer progression, and drug resistance [71,72]. As tubulin is a constituent of all cells, the effects of Les-45, Les-247, or similar compounds on normal cells deserve serious consideration. The cause of the 4-TZD-mediated tubulin loss in the BJ cell line remains to be elucidated, but it might be a result of multiple coordinated changes taking place in the context of alterations in the PPARγ function [73]. Direct measurement of the amount of tubulin in the polymerized and free pools after the Les-45 and Les-247 treatment should resolve these questions. Interestingly, in our study, honokiol alone also increased the β-tubulin and β-actin protein expression in the A549 cells. Our data are consistent with the previously reported research. In in vitro studies, honokiol has been shown to promote the polymerization of the actin and tubulin cytoskeleton, maintaining cellular localization of junction proteins that are responsible for cell polarity [74] and have antineoplastic properties through the induction of cell cycle arrest [75]. Honokiol not only inhibits TNF-α (tumor necrosis factor α)-stimulated NF-κB activation and the STAT3 signaling pathway but may also inhibit the cell cycle through the PI3K/AKT/mTOR pathway by activating PTEN (phosphatase and tensin homolog deleted on chromosome 10) and P21 [76]. The coordinated regulation of cytoskeleton-associated proteins is predominantly influenced by signaling networks, such as the PI3K/AKT pathway, in response to both external and internal stimuli. Onishi et al. [77] highlighted the significance of PI3K/AKT signaling in enhancing the stability of microtubules. Their experiments revealed that the microtubules in fibroblast cells were destabilized by the addition of a pan-PI3K inhibitor LY294002 [77]. Consequently, the modification of microtubule dynamics is thought to support cancer progression, as it promotes uncontrolled motility of cancer cells [78]. In contrast to the aforementioned molecular frameworks, TZD scaffolds are considered potential tubulin inhibitor pharmacophores. Batran et al. [79] showed that a series of 4-phenylcoumarin derivatives containing a thiazolidinone nucleus demonstrated strong tubulin polymerization inhibitory activities. Cell cycle arrest and inhibition of tubulin polymerization in the presence of TZDs have been demonstrated previously in other cellular models [23,80]. For example, a net increase in the percentage of G2/M phase cells upon treatment with TZDs was observed in human T-lymphoblasts (CCRF-CEM) [81] and in colon adenocarcinoma (HCT-15) [82]. Clearly, further work is needed to establish the causal relationship between the cytoskeleton-associated protein expression levels and the potent inhibitory activity against β-tubulin, following treatment with the studied compounds.

## 4. Materials and Methods

### 4.1. Reagents

Trypsin, penicillin, streptomycin, L-glutamine, dimethyl sulfoxide (DMSO), caspase-3 substrate (Ac-DEVD-pNA), resazurin, hydroxyethyl piperazine ethanesulfonic acid (HEPES), sodium chloride (NaCl), 3-[(3-cholamidopropyl)dimethylamino]-1-propanesulfonate hydrate (CHAPS), ethylenediaminetetraacetic acid (EDTA), glycerol, dithiothreitol (DTT), β-nicotinamide adenine (NAD), 2-p-iodophenyl-3-p-nitrophenyl-5-phenyl tetrazolium chloride (INT), 1-methoxyphenazine methosulfate (MPMS), acetic acid, Tween-20, bicinchonic acid (BCA), sodium dodecyl sulfate (SDS), N,N,N′,N′-tetramethylethylenediamine (TEMED), Calcein–AM, and bisBenzimide H 33342 trihydrochloride were purchased from Sigma-Aldrich (St. Louis, MO, USA). Kaighn’s Modification of Ham’s F-12 Medium (F12-K), Dulbecco’s Modified Eagle Medium (DMEM), and phosphate-buffered saline without Ca^2+^ and Mg^2+^ (PBS) were purchased from Corning (Tewksbury, MA, USA). Fetal bovine serum (FBS), radioimmunoprecipitation assay (RIPA) buffer, Universal RNA Purification Kit (E3598-02), and Fast Probe qPCR Master Mix were purchased from EURx (Gdańsk, Poland). Tris base, glycin, and acrylamide/bisacrylamide (30%, 37.5:1 ratio) were purchased from Carl Roth (Karlsruhe, Germany). Lithium lactate was purchased from Thermo Fisher Scientific (Waltham, MA, USA). The High Capacity cDNA—Reverse Transcription Kit and the TaqMan probes corresponding to specific genes encoding ACTB (Hs01060665_g1), GAPDH (Hs02758991_g1), PPARγ (Hs00234592_m1), NF-κB1 (Hs00765730_m1), and NRF2 (Hs00975961_g1)—were purchased from Life Technologies (Forest City, CA, USA). Honokiol was purchased from CaymanChemical (Ann Arbor, MI, USA). Anti-NF-κβ antibodies (1:2000; A10609), anti-Iκβα antibodies (1:2000, A19714), anti-phospho-IκBα antibodies (1:750, AP0707), anti-STAT3 antibodies (1:1000, A1192), anti-GAPDH antibodies (1:100,000, AC033), and anti-β-tubulin antibodies (1:5000, AC021) were purchased from ABclonal Biotech Co., Ltd. (Düsseldorf, Germany). Anti-actin antibodies (1:1500, SC-47778) and Western blotting luminol were purchased from SantaCruz Biotechnology, Inc. (Santa Cruz, CA, USA). Secondary antibodies: anti-mouse (1:3000; 7076P2) or anti-rabbit (1:3000, 7074P2) were purchased from Cell Signaling Technology (Danvers, MA, USA). All stock solutions used in this work were prepared by dissolving the compound powder in DMSO to reach a 100 mM solution. The concentrations obtained were further used to yield 1, 10, 50, and 100 nM and 1, 10, 50, and 100 µM solutions using DMSO as a solvent. The synthesis and physicochemical data of the tested compounds were described previously as Les-45 [23] and Les-247 [24].

### 4.2. Synthesis and Characterization of the Compounds

The complete synthetic protocols and full characterization of the physicochemical properties of the tested compounds were described in [23] for compound Les-45 and in [24] for derivative Les-247.

### 4.3. Cell Culture and Treatment

The human epithelial lung carcinoma A549 (ATCC CCL-185) and the human skin fibroblast cell line BJ (ATCC CRL-2522) were supplied by the American Type Culture Collection (ATCC, distributor: LGC Standards, Łomianki, Poland). The A549 cells were cultured in the F12-K medium, and the BJ cell line was maintained in the DMEM medium. Phenol red-free media containing 4 mM L-glutamine were used in the experiments. Moreover, the media were supplemented with 10% FBS and 0.1% penicillin and streptomycin. The cells were cultured in a humidified atmosphere with 5% CO_2_ at 37 °C until they reached confluence. For resazurin reduction and caspase-3 activity assays, the cells were seeded in 96-well culture plates at a density of 4 × 10^3^ per well (for 24 h and 48 h) and precultured before the experiment for 24 h. Subsequently, the medium was replaced with fresh medium containing increasing concentrations (1, 10, 50, and 100 nM and 1, 10, 50, and 100 µM) of Les-45 or Les-247. For fluorescence-based microscopic observation, the cells were seeded in a ⌀35 mm culture dish at the density of 1 × 10^5^ cells for BJ and 1.2 × 10^5^ cells for A549 per culture dish for 24 h treatment.

### 4.4. Resazurin Reduction Assay

The resazurin reduction viability assay was used to determine the effect of the studied 4-thiazolidinones on cell metabolic activity. Metabolically active cells convert nonfluorescent blue resazurin to fluorescent red resorufin. The assay was performed according to a previously described method [14]. Briefly, a stock solution of resazurin (600 µM in PBS) was aliquoted and stored at 4 C. The aliquots were protected from light and kept in the fridge before use. On the day of analysis, a working solution of 60 µM resazurin was prepared in DMEM (for the BJ cell line) or F12 (for the A549 cell line); both media were supplemented with 1% FBS. The cells were seeded in 96-well plates and incubated with increasing concentrations of the studied compounds for 24 or 48 h at 37 °C. After the 24-h or 48-h treatments of the cells with the tested compounds, the medium was removed and replaced with a new medium containing 1% FBS and 10% resazurin. The fluorescence level was measured after 60 min of incubation at 37 °C using a microplate reader FilterMax F5 Multi Mode (Molecular Devices, Corp., Sunnyvale, CA, USA) at 535 nm excitation and 595 nm emission wavelengths.

### 4.5. LDH Cytotoxicity Assay

Lactate dehydrogenase (LDH) is a soluble cytosolic enzyme that is rapidly released into the culture medium following loss of membrane integrity. This assay measures the reduction in the yellow tetrazolium salt into a red water-soluble formazan-class dye. The amount of formazan correlates directly with the LDH content in the culture supernatant, which is directly proportional to the number of damaged or dead cells. The analysis of the LDH release was performed as in Kaja et al., with modifications [83]. Briefly, a stock solution of buffer A was prepared by dissolving 0.5057 g of iodonitrotetrazolium chloride in 250 mL of 0.2 M Tris-HCl, pH 8.2. The solution was filtered to remove any undissolved particles, aliquoted, and stored frozen at −20 °C. Buffer B was prepared by dissolving 1.1 g of β-nicotinamide adenine dinucleotide sodium salt (NAD) and 8.9 g of lithium L-lactate in 250 mL of 0.2 M Tris-HCl, pH 8.2. This solution was mixed thoroughly, filtered to remove any undissolved particles, aliquoted, and stored frozen at −20 °C. The MPMS supplement was prepared by dissolving 100 mg of methoxyphenazine methosulfate (MPMS) in 1.98 mL of 0.2 M Tris-HCl, pH 8.2, aliquoted, and stored frozen at −20 °C. On the day of analysis, a working solution was prepared by mixing 1.75 mL of buffer A, 1.75 mL of buffer B, and 3 µL of the MPMS supplement. After the 24- or 48-h treatment of the cells with the increasing concentrations of the studied compound, 50 μL of the culture supernatants was transferred to a new 96-well plate. Then, 50 µL of the reaction mixture solution was added and incubated for 60 min in the dark. After 60 min, the reaction was stopped by adding 50 μL of 1 M acetic acid to each well to stabilize the product. The remaining cell plate was frozen at −80 °C and used to measure caspase-3 activity. Absorbance was measured at a wavelength of 450 nm using a FilterMax F5 Multi-Mode microplate reader (Molecular Devices, Corp., Sunnyvale, CA, USA).

### 4.6. Caspase-3 Activity Assay

Caspase-3 activity was used as a marker of cell apoptosis. After the 24- or 48-h treatment of the cells with the tested compounds, the culture plates were unfrozen, and the cells were lysed by adding 50 µL of lysis buffer per well (50 mM HEPES, pH 7.4, 100 mM NaCl, 0.1% CHAPS, 1 mM EDTA, 10% glycerol, and 10 mM DTT) and incubated at 4 °C for 10 min. Then, 50 µL of the working mixture containing the caspase-3 substrate (80 µM) (Ac-DEVD-pNA) and lysis buffer was added. After 30 min, the absorbance of the lysates was measured at 405 nm using a FilterMax F5 Multi-Mode microplate reader (FilterMax F5 Multi-Mode; Molecular Devices, Corp., Sunnyvale, CA, USA).

### 4.7. Hoechst 33342- and Calcein–AM-Based Staining 

The Hoechst 33342 and Calcein–AM staining were used in this study to determine the ability of tested compounds to affect the morphology of cells and nuclei in tested cell lines. The BJ and A549 cells were exposed to 10 μM of Les-45 and Les-247, and the cells were cultured for an additional 24 h. After this period, the cells were washed with PBS and exposed to Hoechst 33342 and Calcein–AM diluted in a medium without FBS at a final concentration of 10 μM and 4 μM, respectively. The cells were incubated for 10 min in an atmosphere of 5% CO_2_ and 37 °C, washed one time in PBS, and visualized using a fluorescence microscope (LSM 700, ZEISS).

### 4.8. Real-Time PCR Analysis of PPARγ, NF-κB, NRF2, and ACTB Genes

This experiment was conducted in accordance with a procedure described previously [84]. For the qPCR assay, BJ or A549 cells were seeded onto 12-well plates and initially cultured for 24 h. Next, the cells were exposed to 10 μM Les-45 or 10 μM Les-247 for 24 h, the samples were collected, and total RNA was extracted from the cells using an RNA isolation kit according to the manufacturer’s instructions (Universal RNA Purification Kit, EURx). The RNA quality and quantity were determined spectrophotometrically at 260 and 280 nm, respectively (NanoDrop ND/1000 UV/Vis, Thermo Fisher, USA). Two-step real-time reverse transcription (RT)PCR was conducted with both the RT reaction and the quantitative PCR (qPCR) run using the CFX Real-Time System (BioRad, Hercules, CA, USA). The RT reaction was carried out at a final volume of 20 μL with 500 ng of the RNA template for BJ and 900 ng of the RNA template for A549 (as a cDNA template) according to the manufacturer’s protocol. The RT reaction products were amplified using the fast probe qPCR Master Mix (EURx) with TaqMan probes as primers for the *PPAR*γ, *NF-κB*, *NRF2*, *ACTB*, and *GADPH* genes in a volume of 20 µL with 1 µL of cDNA. The qPCR was performed with the following reaction parameters: 2 min at 50 °C; 10 min at 95 °C; 15 s at 95 °C = 40 cycles; and 1 min at 60 °C. The threshold value (Ct) for each sample was set during the exponential phase, and the Δ_Ct_ method was used for data analysis. *GAPDH* was used as a reference gene.

### 4.9. Western Blotting

The co-treatment of the cells with the inhibitor of NF-κB (honokiol) was performed to determine the engagement of this pathway in the Les-45 and Les-247 action. Briefly, for the Western blot assay, BJ or A549 cells were seeded onto 6-well plates and initially cultured for 24 h. Subsequently, after 24-h exposure to 10 μM Les-45, 10 μM Les-247, and 15 μM honokiol alone or in co-treatment with the studied compounds, the medium was removed, and the cells were washed once with PBS and lysed using ice-cold RIPA buffer supplemented with protease inhibitors. Protein concentrations in the supernatants were determined using the *BCA* assay with BSA as a standard. From the whole cell lysate, 45 μg of total protein was reconstituted in an appropriate amount of sample buffer, which consisted of 125 mM Tris (pH 6.8), 4% SDS, 25% glycerol, 4 mM EDTA, 20 mM DTT, and 0.01% bromophenol blue. Next, the samples were fractionated by 7.5% SDS-polyacrylamide gel electrophoresis and electrotransferred from polyacrylamide gel to PVDF membranes. Unspecific protein-binding sites were blocked using 1% BSA in TBST and incubated with the following primary antibodies overnight at 4 ◦C: anti-NF-κβ (1:2000; A10609); anti-Iκβα (1:2000, A19714); anti-Phospho-IκBα (1:750); anti-STAT3 (1:1000); anti-β-tubulin (1:5000); anti-actin (1:1500); and anti-GAPDH (1:100,000). Next, the membranes were washed four times in TBST for 10 min and incubated (1 h, RT) with secondary HRP-conjugated anti-mouse (1:3000) or anti-rabbit (1:3000) antibodies. Subsequently, the membranes were washed three times with TBST and visualized by a chemiluminescent substrate (ECL) using the Western Blotting Luminol Reagent (Santa Cruz Biotechnology, Inc., Dallas, TX, USA) and LiCor C-DiGit according to the provided instructions. The densitometric analysis was performed with the GelQuantNET software (version 1.8.2) (BiochemLabSolutions.com, San Francisco, CA, USA). The bands were quantified and normalized to their corresponding GAPDH bands (loading control).

### 4.10. Statistical Analyses

The data were expressed as means ± SD (standard deviations) of six (*n* = 6), twelve (*n* = 12), or three (*n* = 3) repetitions of the experiments (specified in the caption of the graphs). The data were then used in the one-way analysis of variance (ANOVA) with Tukey’s post hoc test using GraphPad Prism 8.0 and denoted as *, **, or *** for *p* < 0.05, *p* < 0.001, or *p* < 0.001, respectively, compared to control cells. The means denoted as # were statistically different at *p* < 0.05 between certain groups (marked in the graphs).

## 5. Conclusions

Our data have shown for the first time that the tested 4-TZDs Les-45 and Les-247 could affect metabolic activity, LDH release, and apoptosis induction in the BJ and A549 cell lines. Moreover, we have proved that these compounds also influenced NF-κB as well as β-tubulin and β-actin, which are engaged in inflammatory and antioxidative stress defenses and cytoskeleton rearrangement. Les-45 decreased the expression of the *NF-κB* gene and protein level (in the BJ and A549 cell lines) and downregulated the expression of *PPARγ* and *NRF2* (in the BJ cell line). In turn, Les-247 increased the expression of *PPARγ* (in the BJ cell line) and decreased *NF-κB* and *NRF2* mRNA (in the BJ and A549 cell lines). Nevertheless, only Les-247 was simultaneously able to reduce the NF-kB protein level in the BJ cells and increase its level in the A549 cell line. This effect was strengthened by honokiol. However, both studied compounds decreased cell metabolism in the BJ and A549 cell lines only at the highest micromolar concentrations; therefore, the use of these compounds as potent anticancer drugs is limited. Further studies on the mechanism underlying the effects of Les-45 and Les-247 in vitro and in vivo are needed.

## Figures and Tables

**Table 1:** IC_50_ values of the studied compounds targeting BJ and A549 cells. The calculated IC_50_ values for the respective time treatments were calculated based on the resazurin reduction measurement results.

IC_50_, µM (M ± SD)				
BJ			A549	
Time Point	Les-45	Les-247	Les-45	Les-247
24 h	>100	>100	50.05 ± 5.14	>100
48 h	>100	>100	33.29 ± 2.03	>100

## Data Availability

Dataset available on request from the authors.

## Abbreviations

4-TZDs—4-thiazolidinones; ACTB—actin beta APS—ammonium persulfate; BCA—bicinchonic acid; BSA—bovine serum albumin; CHAPS—3-[(3-cholamidopropyl)dimethylamino]-1-propanesulfonate hydrate; DMEM—Dulbecco’s Modified Eagle Medium; DMSO—dimethyl sulfoxide; DTT—dithiothreitol; EDTA—ethylenediaminetetraacetic acid; F-12K—Kaighn’s Modification of Ham’s F-12 Medium; GAPDH—glyceraldehyde-3-phosphate dehydrogenase; HEPES—hydroxyethyl piperazine ethanesulfonic acid; INT—2-p-iodophenyl-3-p-nitrophenyl-5-phenyl tetrazolium chloride); IκBα—nuclear factor of kappa light polypeptide gene enhancer in B-cells inhibitor, alpha; LDH—lactate dehydrogenase; Les-45—3-{5-[(Z,2Z)-2-Chloro-3-(4-nitrophenyl)-2-propenylidene]-4-oxo-2-thioxothiazolidin-3-yl}propanoic acid; Les-247—5-[2-Chloro-3-(4-nitrophenyl)-2-propenylidene]-2-(3-hydroxyphenylamino)thiazol-4(5H)-one; MPMS—1-methoxyphenazine methosulfate; NAD—β-nicotinamide adenine; NF-κB—nuclear factor kappa light chain enhancer of activated B cells; NRF2—nuclear factor erythroid 2-related factor 2; P21—cyclin-dependent kinase inhibitor 1; pIKBa—phosphorylated IκBα; PPARγ—peroxisome proliferator-activated receptor gamma; PTEN—phosphatase and tensin homolog deleted on chromosome 10; SDS—sodium dodecyl sulfate; STS—staurosporine; STAT3—signal transducer and activator of transcription 3; T2D—type II diabetes mellitus; TEMED—N,N,N′,N′-tetramethylethylenediamine; TNF-α—tumor necrosis factor alpha.
